# Diverse Physiological Roles of Flavonoids in Plant Environmental Stress Responses and Tolerance

**DOI:** 10.3390/plants11223158

**Published:** 2022-11-18

**Authors:** Aida Shomali, Susmita Das, Namira Arif, Mohammad Sarraf, Noreen Zahra, Vaishali Yadav, Sasan Aliniaeifard, Devendra Kumar Chauhan, Mirza Hasanuzzaman

**Affiliations:** 1 Photosynthesis Laboratory, Department of Horticulture, University of Tehran, Tehran 33916-53755, Iran; 2 Plant Physiology and Biochemistry Laboratory, Department of Botany, University of Calcutta, Kolkata 700019, India; 3 D. D. Pant Interdisciplinary Research Laboratory, Department of Botany, University of Allahabad, Prayagraj 211002, India; 4 Faculty of Environmental Studies, Dehli School of Journalism, University of Delhi, Delhi 110007, India; 5 Department of Horticultural Science, Faculty of Agriculture, Shahid Chamran University of Ahvaz, Ahvaz 61357-43311, Iran; 6 Department of Botany, Government College for Women University, Faisalabad 38000, Pakistan; 7 Department of Botany, Multanimal Modi College Modinagar, Ghaziabad 201204, India; 8 Department of Agronomy, Faculty of Agriculture, Sher-e-Bangla Agricultural University, Sher-e-Bangla Nagar, Dhaka 1207, Bangladesh

**Keywords:** environmental stress, phenolics, photosynthesis apparatus, reactive oxygen species, secondary metabolites

## Abstract

Flavonoids are characterized as the low molecular weight polyphenolic compounds universally distributed in *planta*. They are a chemically varied group of secondary metabolites with a broad range of biological activity. The increasing amount of evidence has demonstrated the various physiological functions of flavonoids in stress response. In this paper, we provide a brief introduction to flavonoids’ biochemistry and biosynthesis. Then, we review the recent findings on the alternation of flavonoid content under different stress conditions to come up with an overall picture of the mechanism of involvement of flavonoids in plants’ response to various abiotic stresses. The participation of flavonoids in antioxidant systems, flavonoid-mediated response to different abiotic stresses, the involvement of flavonoids in stress signaling networks, and the physiological response of plants under stress conditions are discussed in this review. Moreover, molecular and genetic approaches to tailoring flavonoid biosynthesis and regulation under abiotic stress are addressed in this review.

## 1. Introduction

Abiotic stresses affect different aspects of plants’ physiological, biochemical, and molecular status. Nevertheless, through evolution, plants evolved various strategies to overcome stressful conditions by altering their physiological and metabolic pathways. Recent progress in metabolomics enabled the studying of the regulatory roles of metabolites in plants under abiotic stress conditions. It has been well-documented that metabolites play versatile roles in plants’ response to abiotic stresses [1]. Among secondary metabolites, flavonoids are known as “specialized metabolites”. They are low molecular weight polyphenolic compounds that play crucial biological functions in plants and animals [2,3]. More than 6500 flavonoids have been discovered [4]. The US Department of Agriculture has identified several dietary flavonoid subgroups that significantly benefit human health, including anthocyanin, flavonols, flavanones, proanthocyanidins, (iso) flavones, and flavan-3-ols [2]. Most flavonoid compounds prevail in nature as glycosides, and they are soluble in water for the occurrence of sugar and hydroxyl groups in their structure; they are also lipophilic for the presence of isopentyl and methyl groups [5]. Flavonoids are synthesized in specific sites of plant cells, and they control different physiological activities, such as the germination of spores and seeds, the development of aroma and color of flowers, seedlings growth, and also attracting the pollinators toward flower pollen for its dispersion [6,7].

These secondary metabolites participate in defense processes by initiating some biological activities to protect plants when exposed to diverse environmental stresses [8,9]. The accumulation of flavonoids in plants in response to various abiotic stresses, including temperature, heat, freezing, light, UV, nitrogen deficiency, phosphate deficiency, and drought, have been evidenced [10,11]. Flavonoids also play protective roles, including detoxification, allelopathic and antimicrobial effects, phytoalexins, signaling molecules, and signaling UV-filter [12]. Having antioxidant properties, flavonoids can scavenge reactive oxygen species (ROS) under biotic and abiotic stresses [13]. Flavonoids restrict the metabolic activities of enzymes in ROS-generation pathways, thereby stimulating the antioxidant defense system. It is worth mentioning that the diversity in the structure of flavonoids enables them to interact with an immense variety of biomolecules simultaneously [14]. Moreover, several groups of enzymes (isomerases, reductases, and hydroxylases), and Fe^2+^/2-oxoglutarate-dependent dioxygenases act differently in flavonoid biosynthesis, which modifies the fundamental pathway of flavonoid biosynthesis and leads to a different flavonoid subclass [15]. This flexibility at the biosynthesis and functional level makes flavonoids a versatile molecule that can regulate the activities of various enzymes, cell cycles, DNA and protein functions, and lipid peroxidation [14]. However, the biological functions of flavonoids and their role under various environmental stimuli are yet to be unraveled. In the present review, we briefly review the chemistry and biosynthesis of flavonoids in plants to have an insight into their biochemical properties and the mechanism of action in the plant cell. The antioxidative properties of flavonoids and the evidence of the involvement of flavonoids in plants under different abiotic stresses are also discussed. In addition, the underlying mechanisms of the functional roles of flavonoids in shaping a response to different abiotic stresses and their role as signaling molecules in abiotic stress response pathways are probed. Finally, the recent progress in molecular and genetic approaches to tailoring flavonoid regulation under abiotic stresses is discussed. This review provides an overview of the mechanism of plant stress response from the metabolic perspective and enables the assessment of flavonoids as a promising stress marker.

## 2. Chemistry and Biosynthesis of Flavonoids

Flavonoids are the largest group of naturally produced substances, of which more than 9000 phenolic products are detected in *planta* [16]. Flavonoids comprise some primary chemical conformations with three rings of phenols, two of which are joined with the central phenolic ring. The two rings of phenols are associated with 6-carbons independently, and the central ring is associated with the 3-carbons [17]. Various kinds of chemical compounds, as well as derivatives, are synthesized from flavonoids with discrete interchange in their basic chemical constitution. The precursor of flavonoids is flavones, originating in the cell sap of immature plant tissues [18]. Generally, flavonoids are synthesized in the cytosol of the plant cell through different pathways. The synthesis of major classes of flavonoids, including anthocyanins, isoflavonoids, and proanthocyanidins, occur along the general phenylpropanoid and polyketide pathways, transforming phenylalanine into 4-coumaroyl-CoA through cytosolic multienzyme complex (flavonoid metabolon) that is loosely attached to the cytoplasm of the endoplasmic reticulum [19]. The expressions of flavonoid biosynthetic genes such as chalcone synthase (CHS), flavanol synthase (FLS), flavonoid 3′-hydroxylase (F3′H), flavanone 3-hydroxylase (F3-H), and chalcone isomerase (CHI) are mediated by some flavanol regulators, including MYB11/PFG2, MYB12/PFG1, and MYB111/PFG3 [20,21].

**Figure 1:**
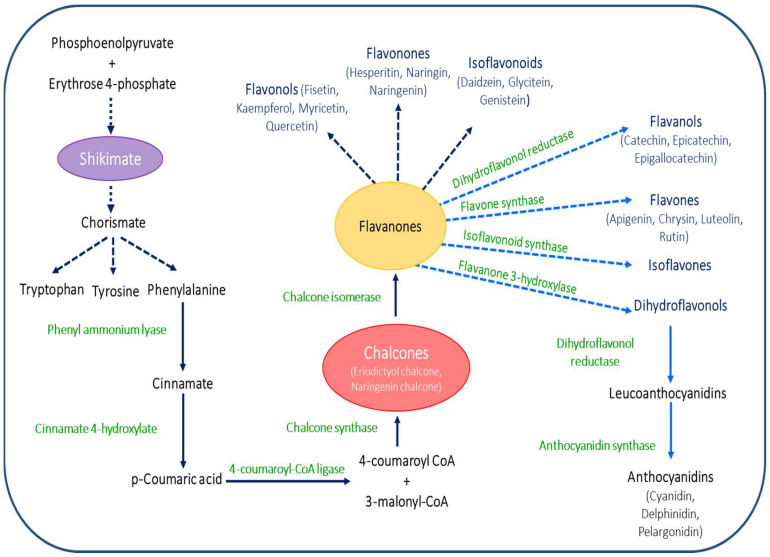
Biochemistry of flavonoids with their various subgroups: Phosphoenolpyruvate and erythrose-4-phosphate are converted to chorismate via the shikimate pathway in seven metabolic stages. Chorismate is the common precursor of three aromatic amino acids viz., tryptophan, tyrosine, and phenylalanine. The enzyme phenyl ammonium lyase (PAL) induces the synthesis of cinnamic acid from phenylalanine, and cinnamate is converted to p-Coumaric acid by the activity of cinnamate 4-hydroxylate. Another enzyme 4-coumaroyl CoA ligase converts p-Coumaric acid into 4-coumaroyl CoA and 3-malonyl CoA, which are responsible for synthesizing chalcones by chalcone synthase activity. Eriodictyol chalcone and naringenin chalcone are the two classes of chalcones. The flavanones are synthesized from chalcones by the activity of chalcone isomerase. There are different subgroups of flavonoids, shown in this diagrammatic representation. Fisetin, kaempferol, myricetin, and quercetin are the types of flavonols. Hesperitin, naringin, and naringenin are the types of flavanones. Some types of isoflavonoids are daidzein, glycitein, and genistein. Flavanols are produced from flavanones by dihydroflavonol reductase activity, and some examples of flavanols are catechin, epicatechin, and epigallocatechin. The enzyme flavone synthase is responsible for the production of flavones from flavanones. Apigenin, chrysin, luteolin, and rutin are some types of flavones. The two other flavonoid subgroups—isoflavones and dihydroflavonols—are synthesized by the activity of isoflavonoid synthase and flavanone 3-hydroxylase, respectively. Dihydroflavonol reductase converts dihydroflavonols into leucoanthocyanidins, which are converted into anthocyanidins by the anthocyanidin synthase enzyme. Cyanidin, delphinidin, and pelargonidin are some types of anthocyanidins.

In plants, flavonoid accumulation depends upon the modulation of the expression of genes related to the flavonoid biosynthetic pathway [22,23,24]. The phenylpropanoid pathway gives p-coumaroyl-CoA. This pathway starts from the aromatic amino acids phenylalanine and tyrosine, produced by the shikimate pathway (Figure 1). The flavylium ion is known as the core of the flavonoids’ biosynthesis pathways, the upstream of which is three molecules of malonyl-CoA and one molecule of 4-coumaroyl-CoA [25]. Furthermore, CHS and CHI enzymes are involved in the two-step condensation, which yields in naringenin (flavanone) [26].

**Figure 2:**
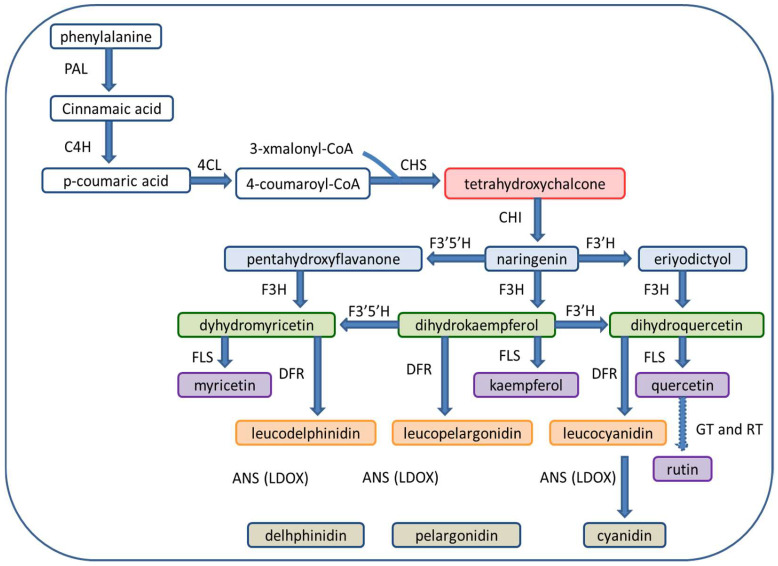
Basic pathway of flavonoid biosynthesis in plants. Abbreviations:—PAL, phenylalanine ammonia lyase; C4H, cinnamate 4-hydroxylase; 4CL, 4-Coumaroyl CoA ligase; CHS, chalcone synthase; CHI, chalcone isomerase; F3H, flavanone 3-hydroxylase; F3′H, flavonoid 30-hydroxylase; F3′5′H, Flavonoid 3050-hydroxylase; FLS, flavonol synthase; DFR, dihydroflavonol 4-reductase; ANS, anthocyanidin synthase; LDOX, leucoanthocyanidin dioxygenase; GT, glucosyltransferase; RT, rhamnosyltransferase.

The oxidation of naringenin by F3-H produces the dihydrokaempferol that could further be hydroxylated on the 3′ or 5′ end of the B-ring, by F3′H or flavonoid F3′5′H, producing dihydroquercetin (dihydroflavonols). F3′H or F3′5′H could directly hydroxylate naringenin to produce eriodictyol and pentahydroxy-flavanone (flavanone); they are further hydroxylated to dihydroquercetin and dihydromyricetin. The synthesized dihydroflavonols are further transformed into flavonols, flavan-3,4,-diols, and anthocyanidins by reactions catalyzed by FLS, DFR, and LDOX. The DFR changed dihydroquercetin, dihydrokaempferol, and dihydromyricetin to leucocyanidin, leucopelargonidin, and leucodelphinidin (flavan-3,4,-diols); further catalyzation by LDOX causes their oxidation to cyanidin (red-magenta anthocyanidin), pelargonidin, and delphinidin (anthocyanidins) [27] Figure 2.

The flavonoid-synthesizing enzymes have a loose attachment with the endoplasmic reticulum (ER). Other enzymes in flavonoid biosynthesis pathways are linked with the membranes of the vacuole, plastids, and nucleus [28]. Moreover, a supermolecular network via protein–protein association between flavonoid biosynthesizing enzymes with the ER membrane has been reported [12]; those flavonoid-synthesizing enzymes are classified in different enzymatic groups, including glycosyl transferases, cytochromes P450 (cyt P450), and 2-oxoglutarate dioxygenases (2-OGD) [29]. The important flavonoid subgroups found in plants are anthocyanins, chalcones, flavonols, flavanols, flavanonols, flavanones, flavones, and isoflavonoids [7] Figure 1. Depending upon the attachment of carbon rings, the flavonoids’ subgroups were recognized, such as neoflavonoids or 4-benzopyrans, isoflavonoids or 3-benzopyrans, flavonoids or 2-phenylbenzopyrans, etc. [12].

Several studies noted the localization of FLS 1, CHS, and CHI in nuclei of *Arabidopsis* [30,31,32]. However, most of the flavonoids accumulated in the cytoplasm are then possibly moved into the vacuole via an autophagic mechanism [33] and grape-vesicle, concerning a GST and two multidrug and toxic compound extrusion-type transporters (anthoMATEs) [34,35]. This transformative nature and reactivity may underly flavonoids’ versatility in abiotic stress response in plants.

## 3. Antioxidant Properties of Flavonoids

**Figure 3:**
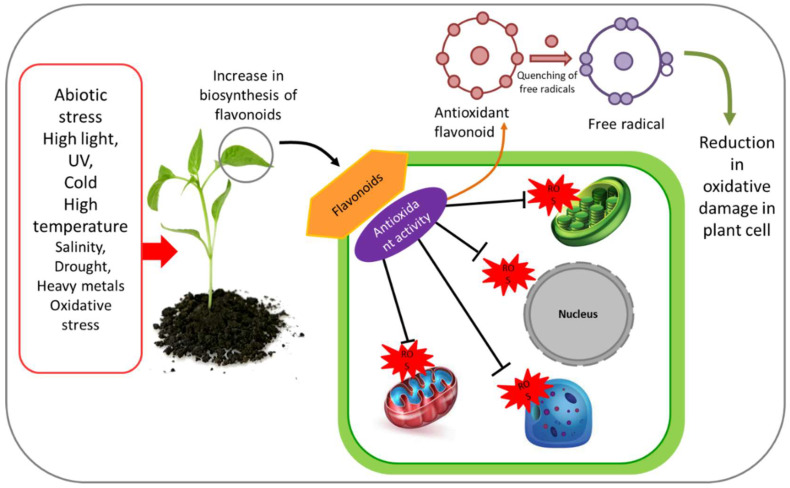
Antioxidant activities of flavonoids under abiotic stress. Following abiotic stress in plants, biosynthesis and accumulation of flavonoids takes part in reactive oxygen species (ROS) scavenging in different plant cell organelles and reduces oxidative damage in plant cells.

Flavonoids act as antioxidant in plants and provide protection against various environmental stresses (Figure 3; Table 1). A consequence of abiotic stresses is the production of harmful ROSs [36,37]. These are known as highly reactive superoxide anion radical (O_2_^•−^), singlet oxygen (O_2_), hydrogen peroxide (H_2_O_2_), and hydroxyl radical (•OH). Among the highly reactive and dominant ROSs are H_2_O_2_ and O_2_^•−^. These ROSs cause oxidative stress that occurs as a consequence of disturbance in maintaining homeostasis between ROS production and endogenous antioxidant defense mechanism [37]. During environmental stress, ROSs act as oxidants of the DNA, proteins, carbohydrate, and lipids and eventually cause damage to plant cells [38]. To cope with oxidative damage, plants produce antioxidant enzymes (e.g., SOD, CAT, ascorbate peroxidase (APX), glutathione peroxidase (GPX), glutathione reductase (GR), etc.). However, under extreme environmental stress conditions, the production of antioxidants in plants cannot keep pace with the magnitude of the oxidation, leading to increased ROS content in the cell [39]. Under such circumstances, the antioxidant properties of flavonoids help plants to counterbalance the excessive ROS production and repair the damage caused by them [40,41]. Flavonoids are a large class of secondary metabolites, and several pieces of evidence confirm the hypothesis of their antioxidant functions in higher plants under a range of environmental stresses [42,43]. With potent antioxidant properties, flavonoids help plants to cope with oxidative stresses by quenching free radicals, thereby protecting plants from cellular peroxidation [44]. The suppression of ROS generation by flavonoids occurs through the four following pathways: (i) restriction of singlet oxygen, (ii) inhibition of ROS-producing enzymes (cyclooxygenase, lipoxygenase, monooxygenase, and xanthine oxidase), (iii) chelation of transition metal ions, and (iv) recycling of other antioxidants [45,46].

Flavonoids occur abundantly in different parts of plants, mostly in the vacuole and chloroplasts of the mesophyll cells, and are also found consistently in the subcellular sites to function as ROS-quenchers [47,48]. During stress, the presence of flavonoids in the vacuole helps detoxification of H_2_O_2_ molecules, which are generally released from the chloroplast [49]. Several studies have shown the antioxidant properties of flavonoids through different actions. The general modes of action of flavonoids against stresses are (i) quenching of free radical molecules, (ii) metal chelation, (iii) interfering with the enzymes related to free radical generation, and (iv) activation of plants’ natural antioxidant enzymes [50]. Flavonoids are directly involved in scavenging ROS. Having chelating properties, flavonoids take part in the chelation of free radicals by donating a hydrogen atom or by single-electron transfer, as well as through the chelation of transition metal elements to prevent free-radical formation [51]. Additionally, flavonoids function as an internal antioxidant enzyme by hindering free-radical triggering enzymes, for instance, xanthine oxidase, lipoxygenase, protein kinase C, cyclooxygenase, microsomal monooxygenase, mitochondrial succinoxidase, and NADPH oxidase [50,52].

Numerous abiotic stresses trigger highly hydroxylated flavonoids. Under such a state, the stronger scavenging function occurs by the activity of an extra free hydroxyl radical (–OH) on the C-30 position of the B-ring [42,53]. A study on soybean seedlings treated with lanthanum demonstrated flavonoid potential for scavenging O_2_ and ·OH [54] by decreasing the MDA concentration and maintaining standard plasma membrane permeability. Quercetin 3-O- and luteolin 7-O-glycosides, having a catechol group (ortho-dihydroxy B-ring substitution) in the B-ring of the flavonoid skeleton, show considerable antioxidant activity in plant cells [53]. Moreover, quercetin derivatives protect chloroplast damage from the singlet oxygen induced by high light in *Phillyrea latifolia* leaves [48]. Similarly, kaempferol, a monohydroxy B-ring flavanol, also showed antioxidant properties under light irradiance [55]. It has been observed through studies that in most cases, quercetin derivatives are more efficient than monohydroxy B-ring, particularly during complex formation with ions of Cu and Fe. They are also found to be involved in inhibiting ROS production by the Fenton reaction [56] and suppressing generated ROS, as well as equipping plants with versatile compounds to cope with environmental stresses.

**Table 1:** Antioxidative role of flavonoids in plant response to abiotic stress.

Abiotic Stress	Plant Species	Antioxidant Response of Flavonoids	References
UV-B radiation	*Medicago sativa*	Increased content of flavonoid compound induces enhanced antioxidant capacity of the plant.	[57]
UV-B radiation	*Kalanchoe pinnata*	Increases total flavonoid and quercitrin content, which have antioxidant properties to protect the plant.	[58]
UV-B stress and drought	*Populus tremula × P. tremuloides*	Transgenic line of poplar with high proanthocyanidins content displayed lower hydrogen peroxide content.	[59]
Salinity	*Zea maize*	Improved plant performance under salt stress through antioxidant activities.	[60]
Salinity	*Arabidopsis thaliana*	CrUGT87A1, a UDP-sugar glycosyltransferases (UGTs) gene, improved salt tolerance by increasing antioxidant capacity resulting from the accumulation of flavonoids.	[61]
Salinity	*Amaranthus tricolor*	Increases flavonoid content, which showed the potent antioxidant activity in scavenging ROS.	[62]
Salinity	*Amaranthus lividus*	Increases flavonoid content and the antioxidant capacity of leaves, total flavonoid content scavenged ROS.	[63]
Water stress	*Chrysanthemum morifoilum*	Increases flavonoids (rutin, quercetin, apigenin, and luteolin) and enhanced antioxidant activity.	[64]
Drought	*Arabidopsis thaliana*	Increase in total flavonoid content followed by an increase in antioxidant activity.	[65]
Drought	*Cistus clusii*	prevented oxidative damage.	[66]
Drought	*Swingle citrumelo*	Proline accumulation was concomitant with an increase in antioxidant activity.	[67]
Temperature stress	*Solanum viarum Dunal*	Flavonoids inhibited ROS-mediated oxidative damage.	[68]
Heat and salinity	*Solanum Lycopersicon*	Lower antioxidative damage was observed following a high accumulation of flavonols.	[69]
Cadmium stress	*Trigonella foenum-graecum*	H_2_S-induced polyamines accumulation was concomitant with an increase in ROS-detoxification capacity.	[70]
Cadmium stress	*Solanum Lycopersicon*	Nitric oxide-induced increase in flavonols resulted in improved antioxidant capacity.	[71]
Lead stress	*Tritium aestivum*	Accumulation of proline was concomitant with a lower level of lipid peroxidation.	[72]

## 4. Flavonoids-Mediated Defenses against Abiotic Stress

**Figure 4:**
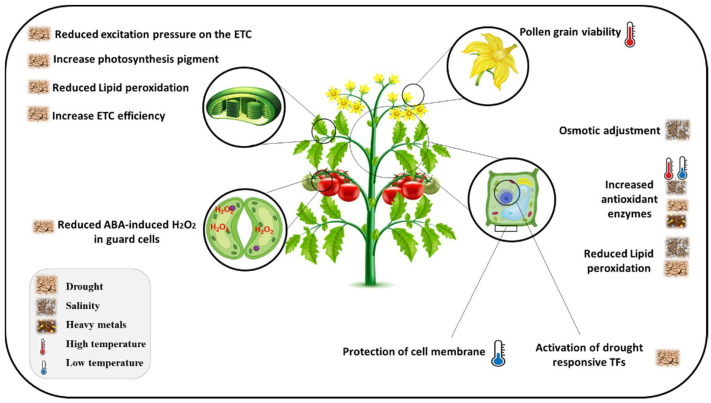
The role of flavonoids in abiotic stress response in plants. Modulation of excitation pressure, increase in photosynthesis pigments, reduced lipid peroxidation in the thylakoid membrane, enhancement of osmotic adjustment, reduction of ABA-induced ROS in guard cells, and activation of stress-responsive transcription factors under osmotic stress. Improving pollen grain viability under high temperature and protection of cell viability under cold temperature.

To survive under abiotic stresses, plants adopt different strategies at molecular [73], metabolomic [74], physiological, and morphological levels. The common aftermath of abiotic stress is ROS production, accumulation, and signaling. Accordingly, the scavenging of ROSs is an inevitable part of shaping a response to abiotic stress. Flavonoids are secondary metabolites with antioxidant properties that play an efficient role in ROS scavenging and the prevention of ROS generation [41]. Besides antioxidant properties, different mechanisms and sites of action have been proposed for flavonoids in plants’ stress tolerance (Figure 4; Table 2 and Table 3). Flavonoids are mostly species-specific compounds [3,4], and their biosynthesis is dependent on the plant species, developmental stage, and the nature of the stresses [12,75]. Many studies have reported the alternation in the level of flavonoid contents under different stress conditions [76].

**Table 2:** Differential response of flavonoids under different abiotic stress.

Abiotic Stress	Concentration/Levels	Duration of Stress	Plant Species	Flavonoids Level under Stress	References
Salinity	50 and 100 mM NaCl	35 days	*Amaranthus lividus*	An increase was observed in total flavonoid content by 31%.	[77]
Salinity	200 mM NaCl	3 weeks	*Apocynum venetum* L.	The total flavonoid content and dihydroquercetin decreased by 20.46% to 23.08%, but an increase in flavonols (quercetin and kaempferol) by 1.6-fold and 2.2-fold was detected in comparison to control.	[78]
Drought	Stop watering	5 days	*Arabidopsis thaliana* L.	Quercetin 3-O-glucoside and cyanidin 3-O-glucoside exhibited approximately10-fold higher activity than kaempferol 3-O-glucoside, whereas a slight reduction in total flavonoid content was observed.	[79]
Drought	Osmotic potential of 0.49 MPa	48 h	*Triticum aestivum* L.	Significant increase in total flavonoid content was detected by 143% in cultivars aikang 58 compared with Chinese spring (115%).	[80]
Drought	Soil water content 25% (±2.5%)	At three-leaf seedling stage	*Zea mays* L.	Flavonol in guard cells was observed 1.7-fold higher compared to control.	[81]
Copper	200 mg L^−1^	35 days	*Belamcanda chinensis*	Increased generation of 11 kinds of flavonoids.	[82]
Copper and Zinc	200–500 ppm	28 days	*(Lycopersicon esculentum Mill*	Accumulation of flavonoids increased (1.44, 0.93 mg QE/g DW) compared to the control (0.18, 0.13 mg QE/g DW) in roots and leaves, respectively.	[83]
UV-B and drought	40% drought-stressed	8 weeks	*Ligustrum vulgare* L.	Increases in the biosynthesis of quercetin-3-O-rutinoside, luteolin 7-O-glucoside, and echinacosid were observed.	[53]
Extreme temperature and high CO_2_ levels	Light intensity 700 PAR and ambient CO_2_ (400 µmol mol^−1^)	35–39 days	*Lactuca sativa* L.	Increased accumulation of quercetin-3-O-glucoside, quercetin-3-O-glucuronide, luteolin7-O-glucoside, cyanidin derivatives (61%), and cyanidin-3-O-glucoside (28%), while lower accumulations of kaempferol, myricetin, quercitrin (99–94%), and rutin were found under high light condition. Total flavonoid content increased by 7.5-fold in comparison to control.	[84]

**Table 3:** Functional role of flavonoids in plants’ response to abiotic stresses.

Stress	Stress Level	Duration of Stress	Plant Species	Flavonoids Modulation	Function of Flavonoids	Reference
Drought	Drought (mild drought stress)	24 h	Tea (*Camellia sinensis*)	Accumulation phenylalanine ammonia-lyase (PAL), cinnamic acid 4-hydroxylase (C4H), 4-coumarateCoA ligase (4CL), chalcone synthase (CHS), and dihydrofavonol 4-reductase (DFR).	Increase in flavonoid content was concomitant with stress tolerance in plant.	[85]
Drought	15–25% of soil water-holding capacity	8 days	Tea (*C. sinensis*)	Accumulation of endogenous flavonoids, including: C4H, CHS, F3′5′H, F3H, kaempferol, quercetin, and myricetin triggered by fulvic acid.	Increase in flavonoid content took part in improved tolerance of plants against drought.	[86]
Drought	8% PEG 6000	7 days	Maize (*Zea mays*) Pigeon pea (*Cajanus cajan*)	Accumulation of endogenous flavonoids, including: genistein, genistin, and pterostilbene.	ABA and CcMYB114 improve drought tolerance by regulating the accumulation of flavonoids.	[81,87]
Drought	Stopped watering	3 weeks	*Arabidopsis* (*A. thaliana*)	Accumulation of endogenous flavonoids triggered by ectopic expression of Arabidopsis glycosyltransferase gene (UGT76E11).	Activation of stress-related transcription factors.	[88]
Salt	300 mM NaCl	14 days	*Arabidopsis* (*A. thaliana)*	Accumulation of endogenous flavonoids including: chalcone, dihydrokaempferole, and quercetin.	Act in MYB111-regulated salt stress response.	[89]
Salt	100, 150, and 200 mM NaCl	19 days	Maize (*Z. mays*)	Exogenous application of α-tocopherol in combination with selenium (Na_2_SeO_4_ (0.5 mM) + a-tocopherol (200 ppm)).	Improved plant performance under salt stress through antioxidant defense.	[60]
Salt	150 mM NaCl	5 days	Tomato (*Solanum Lycopersicon* L.)	Exogenous application of vanillic acid (4- hydroxy-3-methoxy benzoic acid) (50 μM).	Increase in the activity of AsA-GSH cycle and glyoxalase system and a further increase in accumulation of osmolytes. Improved K^+^ accumulation and restricted Na^+^ accumulation. Increase in superoxide dismutase (SOD), catalase (CAT), and ascorbic acid (AsA).	[90]
Salt	100 mM NaCl	8 days	Bean (*Phaseolus vulgaris*)	Exogenous application of naringenin (0.1–0.4 mM).	Regulation of cellular redox, chloroplast antioxidant system, and photosynthesis.	[91]
Heavy metals	150 mg L^−1^ of Pb2 þ (which corresponds to 724 μM Pb(NO_3_)_2_)	Incubated for 2 h	Lupin	Incubation of seedlings with catechin before exposure to lead stress (5, 10, and 20 μg mL^−1^ of catechin equivalents).	Increased root growth and reduced accumulation of ROS, lipid peroxidation, and cell death.	[92]
Heavy metals	Wastewater	100 days	Lettuce and turnip	Accumulation of endogenous flavonoids, including putrescine and spermidine.	Counteract the oxidative stress.	[93]
High temperature	37 °C (day), 25 °C (night)	During growth period	Tomato (*Solanum Lycopersicon* L.)	Accumulation of endogenous flavonoids.	Reducing the abundance of ROS, enhancing fertility.	[94]
High temperature	Moderate (36 °C/24 °C day/night) or severe (42 °C/26 °C day/night)	During the growth period since the pod’s color changed to an individual level	Soybean (*Glycine max*)	Accumulation of endogenous flavonoids, including tocopherols, flavonoids, phenylpropanoids, and ascorbate precursors.	Scavenging of heat-induced ROS damage during seed maturity.	[95]
Air pollutant	Sulfur dioxide (SO_2_), NO_2_, carbon monoxide (CO), hydrocarbons (HC), and airborne particulate material (APM)	During growth period	*Spartium junceum* L., *Lagerstroemia indica* L., *Th uja orientalis* L., and *Petunia hybrida* L. w	Accumulation of endogenous flavonoids.	Reduced ROS accumulation in pollen grain and improved development of pollen tube and germination.	[96]
Air pollutant	O_3_ stress (300 nL L^−1^)	6 h	*Medicago truncatula*	Accumulation of endogenous phenolic compounds.	Phenols were oxidized red/purple pigments and resulted in the accumulation of antioxidant compounds.	[97]

### 4.1. Drought and Salinity

Drought is known as the most important physical stress of terrestrial ecosystems [98]. Therefore, different research has dealt with drought by studying either agricultural water saving and water reuse [99] or by considering plants’ physiological adaption to the limited water supply [100]. Salinity occurs when the number of nutrient elements exceeds a species-specific threshold and threatens plant productivity [101].

Alternation in gene expression, metabolic modifications, osmotic adjustment [102], regulation of stomatal movement [103,104], and adjustment of growth and development are among drought-adaption strategies that are activated in plants to maintain the water balance [105,106,107].

A study on tea plants revealed that under drought stress, the expression of the genes related to flavonoid biosynthesis, including CHS, dihydrofavonol 4-reductase (DFR), Leucoanthocyanidin reductase (LAR), and leucoanthocyanidin dioxygenase (ANS), was decreased in the early stages of the drought but subsequently increased by continuous drought stress [108]. The significant upregulation of flavonoid biosynthesis genes (phenylalanine ammonia-lyase (PAL), cinnamic acid 4-hydroxylase (C4H), 4-coumarateCoA ligase (4CL), CHS, and Dihydrofavonol 4-reductase (DFR)) was demonstrated by another study on tea plant under drought condition [85]. The positive effect of fulvic acid in improving the drought resistance of tea was shown to be related to its role in activating flavonoid biosynthesis pathway genes [86]. Another piece of evidence of the involvement of flavonoids in drought stress response was reported in *Arabidopsis* by indicating that a highly drought-induced gene, CYTOCHROME P450, was involved in the upregulation of antioxidant flavonoids genes in *Arabidopsis* [65]. Coexpression of flavonoid biosynthesis genes and drought-induced genes, as well as upregulation of flavonoid biosynthesis genes under drought stress, accounts for the involvement of flavonoids in drought stress responses. The mechanism of flavonoid action under drought has been proposed by previous studies and portrays several interlinked pathways, including antioxidant properties, signaling components, osmotic adjustment, stomatal movement, and photosynthesis regulation.

In plants, the role of flavonoids in response to salt stress has also been proposed. A transgenic line of *Arabidopsis*, UGT76E11, that overaccumulates flavonoids exhibited a high antioxidant capacity, reduced ROS accumulation, and enhanced NaCl and mannitol stress resistance [88]. A genotype-dependent manner was detected in the accumulation of flavonoids upon short-term or long-term salt stress in two Cardoon genotypes. The genotype “Bianco Avorio” showed a constant increase in flavonoid content in response to both short- and long-term stresses, while in “Spagnolo”, only long-term salt stress triggered flavonoids accumulation [109]. The *Arabidopsis* MYB transcription factor, *MYB111*, regulates salt stress responses, as a reduction in *MYB111* is significantly linked with reduced salt tolerance in *Arabidopsis*. An increase in flavonoid biosynthesis was associated with *MYB111* overexpression, suggesting that flavonoids act in *MYB111*-regulated response to salt stress tolerance. To test the hypothesis, the researchers examined the effect of exogenous bioflavonoids such as chalcone, dihydrokaempferole, and quercetin on salt-stressed *Arabidopsis* plants. They found that these isoflavones rescued the decreased salt tolerance in *MYB111* mutants [89]. Coexpression network analysis of salt-tolerant wild soybean revealed that the mechanism of class B heat shock factor, HSFB2b, in soybean response to salinity stress partially underlies its role in activating a subset of genes related to flavonoid biosynthesis [110]. This part explains the mechanism of flavonoid involvement in drought stress response in plants.

A primary consequence of drought stress is oxidative damage. Antioxidant systems take part in the amelioration of oxidative damage through the activation of enzymatic or nonenzymatic antioxidants that provide effective scavenging of ROS [111]. Flavonoids are among nonenzymatic antioxidants that improve plants’ fitness to drought stress.

An increase in the antioxidative defensive mechanism as a result of selenium and a-tocopherol was the result of enhancement in the production of phenolics and flavonoid content in maize plants under salt stress, signifying the antioxidative role of flavonols in the salt stress response pathway [60]. Exogenous application of vanillic acid took part in osmolyte accumulation, regulation of ion uptake, and augmentation of superoxide dismutase (SOD), catalase (CAT), and ascorbic acid (AsA) under salt stress [90].

Another role of flavonoids is improving the plants’ adaption to drought by regulating stomatal movements. It was revealed that flavanols hinder ABA-induced hydrogen peroxide (H_2_O_2_) accumulation in stomata guard cells of *Arabidopsis* [112]. It was also found that the accumulation of flavonols in stomata guard cells was highly induced by drought, and the accumulation of flavonols was higher in a drought-overly-insensitive (doi57) mutant compared with the wild type, which was associated with a relatively lower accumulation of H_2_O_2_ in stomata guard cells of doi57 [81]. In a study on pigeon pea, the accumulation of flavonoids (genistein, genistin, and pterostilbene) was accompanied by the initiation of stomatal closure by ABA treatment under drought [87]. Gene coexpression networks in sea buckthorn revealed that ABA and flavonoid signaling crosstalk determines the levels of drought resistance among different subspecies [113]. Unraveling the metabolic signature of *Brassica napus* in response to ABA suggested a role for flavonols in stomatal movement under drought stress. Further examination showed that the exogenous application of 1 µM quercetin resulted in a slight increase in the stomatal aperture of *B. napus* [114].

A role as a signaling molecule for flavonols has also been suggested [88,115]. Increased transcription of stress-related genes in the UGT76E11 transgenic line of *Arabidopsis* was featured by flavonols overaccumulation, suggesting a role for flavonols as a signaling molecule that activates stress-related transcription factors [88]. A study on *Arabidopsis* indicated that ectopic expression of a grape Basic helix-loop-helix (bHLH) transcription factor gene, VvbHLH1, increased the accumulation of flavonoids. Authors suggested that overexpression of VvbHLH1 resulted in adaption to salt and drought stress by upregulation of genes involved in the ABA biosynthesis pathway, which further increases the generation of signaling molecules and the expression of stress-tolerance genes [116].

Flavonoid accumulation improved photosynthesis by decreasing lipid peroxidation and lowering excitation pressure and loss of energy through nonphotochemical quenching [117]. Therefore, flavonoids take part in drought stress responses at different levels, including signal transduction, regulation of gene expression, ROS scavenging, stomatal movements, and retention of photosynthetic system functionality, and eventually improve plants’ performance under drought stress conditions. Microarray analysis indicated that upregulation of a gene encodes chalcone isomerase2 (OsCHI2) under drought and salt stress. The OsCHI2 is responsible for increasing the transcripts of structural genes related to the flavonoid’s biosynthesis pathway. Rd29A::OsCHI2 transgenic rice plants exhibited prolonged photosynthesis activity under drought and salinity stress. An increase in relative water content, photosynthetic pigments, and proline with reduced relative electrolyte leakage and malondialdehyde content detected in plants were suggested as the mechanisms by which flavonoids take part in the regulation of photosynthesis activity under drought and salinity [118]. Another study proposed that the positive effect of mild NaCl treatment on net photosynthesis (P_n_) and quantum yield efficiency of electron transfer (F_V_/F_M_) was the result of an increase in the total flavanols content of *Tetrastigma hemsleyanum* [119]. Alleviation of the effect of salinity on cellular redox, chloroplast antioxidant system, and photosynthetic activity is indicated by applying exogenous naringenin on bean plants (*Phaseolus vulgaris*) under salt stress [91]. A genotype-dependent response of photosynthesis to salt stress was detected in two Paulownia genotypes. Further investigation that displayed different capacities for the accumulation of flavonoids in *Paulownia tomentosa* × *fortune* (TF) compared with *Paulownia elongata* × *elongata* (EE) underlies the variation in their potential to respond to salt stress. The genotype with a higher capacity of flavonoid accumulation (TF) showed higher resilience of photosynthesis apparatus, indicated by higher F_V_/F_M_ and higher QA^-^ reoxidation compared to EE [120].

Moreover, flavonoids improve plants’ resistance to drought and salt stress by preventing oxidative processes, maintaining a fine-tuned oxidation/redox potential, osmotic regulation, and improving photosynthesis efficiency. Therefore, the accumulation of flavonols in plants under salt stress favors plants’ resilience to drought and salt from both molecular and physiological aspects.

### 4.2. Toxic Metal/Metalloids

Flavonoids, as a versatile compound in abiotic stress alleviation, also take part in response to heavy metal stress. The concomitant increase in flavonoids with an increase in the concentration of heavy metals in plant tissue suggested an antioxidative role for flavonoids in alleviating heavy metal stress in plants [121,122,123]. Moreover, the phytoremediation capacity of *N. biserrate* was concluded to be the result of a high accumulation of myricetin and kaempferol in its tissue when grown in heavy metal-contaminated soils [124]. Preincubation of lupin seedlings exposed to lead stress for 48 h with flavonoids attenuated the adverse effects of lead stress. Increased root growth, reduced accumulation of ROS, lipid peroxidation, and cell death were detected in flavonoid-incubated plants compared to control under lead toxicity. To answer the query related to the effect of flavonoids on the removal of excess lead due to its antioxidant properties, the capacity of root extracts to scavenge 1-diphenyl-2-picrylhydrazyl (DPPH) was investigated and confirmed the antioxidative role of flavonoids in lead stress-exposed plants [92]. Flavonoids enhanced the tolerance of *Avicennia marina* to Cd. However, flavonoids showed no influence on the uptake of Cd in root cell walls since the exposure of roots to ion transport inhibitor (LaCl_3_) evidenced the facilitation of Cd transport in roots, indicating that flavonoids have a significant stimulative effect on symplastic transport of Cd in roots, and Ca-channel was not the unique means of symplastic transport for Cd absorption. Flavonoids facilitate symplastic transport when roots take up Cd but do not affect apoplastic transport [125]. According to the existing literature, the antioxidative role of flavonoids is the only mechanism of heavy metal stress alleviation in plants that has been taken into consideration thus far. Nevertheless, there are a limited number of reports on the metal-chelation properties of flavonoids. In a study on *Fagopyrum esculentum* by Moench, the role of salicylic acid in alleviating Cd stress was attributed to its effect on the enhancement of the metal-chelation properties. Heavy-metal chelation properties have also been assigned to plant-based natural flavanols in a study on the effect of lead poisoning in mice [126].

### 4.3. Extreme Temperature

Low temperature upregulates the expression of flavonoids’ biosynthetic genes and increases the content of flavonoids in plant tissue in a species-dependent manner [127,128]. Flavonoids were also introduced as the potential biomarkers for cold stress in barley [129]. Reportedly, anthocyanin synthesis plays an essential role in cold stress tolerance in *B. rapa* since the expression of anthocyanidin synthase (BrANS) genes was sturdily related to cold-stress tolerance [130], whereas knock-out mutation of PRODUCTION OF ANTHOCYANIN PIGMENT 1 (PAP1) MYB transcription factor depicts impaired leaf-freezing tolerance in *Arabidopsis* [128].

The role of anthocyanin and other flavonoids in the tolerance of *Arabidopsis* to cold stress has been reported. Nevertheless, the precise causal relationship between flavonoids and cold stress tolerance was not proposed [131]. Other researchers have studied the tolerance of *Arabidopsis* against freezing to initiate the stress linked with apoplastic ice crystal formation at subzero temperatures. Their investigation indicated minor effects of flavonoids on primary metabolism. They also refuted the possibility of involvement of flavonoids in the modification of phytohormones’ balance or stabilization of proteins as a possible function of flavonoids in chilling stress. This was because plant growth, development, and primary metabolism were unaltered in all flavonoid biosynthesis mutants used in their study. Instead, they approved a previously proposed role for flavonoids in freezing tolerance because flavonoids take part in the protection of cell membranes and proteins against cold stress since flavonoids-mediated partition and stability of plants’ membranes have been evidenced [132]. They also proposed that the redundancy of flavonoid structures allows the deficiency of flavanols or anthocyanins to be compensated by other flavonoid compound classes [133]. A close association between cold stress tolerance and expression of dihydroflavonol 4-reductase (DFR) genes is known to be another essential function in the flavonoid biosynthetic pathway. This association proposed that the BrDFR gene is a useful resource for molecular breeding of freezing stress-resistant Brassica crops [134].

In addition, a role for flavonoids as an osmoticum has been proposed in a study on apple leaves exposed to cold temperatures. Although the role of anthocyanin in the osmotic adjustment of apple leaves has been proved by their study, due to metabolic costliness relative to other osmolytes and low concentrations, it is unlikely that they solely take part in osmoregulation [135]. Moreover, in a study on *Liriope spicata*, it was revealed that genes and metabolites involved in the flavonoid pathway had a synergist role in osmoregulation under freezing stress [136].

Interactions between light and cold stress have been depicted by several studies [137]. A study on the interactive pathway of blue light signaling with cold stress response depicted the dependency of anthocyanin biosynthesis on the expression of cold-stress-responsive genes affected by blue light signaling [137,138]. The role of light intensity and spectra on flavonoids, particularly anthocyanin, has recently attracted attention and was briefly discussed in the section on light stress.

The comparison between pepper plants (*Capsicum annuum* L.) incubated by *Penicillium resedanum* with nonincubated plants showed that tolerance to high temperature was associated with the uplift in amino acid and the production of flavonoids in high quantities [139]. On the other hand, transcriptomic analysis of eggplants under high temperature displayed downregulation of genes in the anthocyanin biosynthetic pathway of eggplant [140]. The role of flavonoids in enhancing the fertility of tomatoes under high temperatures was investigated. Studying anthocyanin-reduced (*are*) tomato mutants demonstrated that flavanols ameliorated the adverse effects of high temperature by reducing the abundance of ROS [94]. The ROS scavenging role of flavonoids as the potential function of flavonoids in attenuation of heat stress was also reported in heat-stressed soybean seeds. The authors proposed that higher concentrations of flavonoids, ascorbate precursors, and tocopherols alleviated heat stress damage during seed maturity via scavenging heat-induced ROS damage [95]. In contrast, the reduction of flavonoids in response to high temperatures has also been reported, suggesting a negative role for flavonoids in plants’ fitness to high temperatures [141,142]. However, combined heat and drought stress led to increased flavonols content in *Quercus ilex* L. [143].

In conclusion, an increase in flavonoid content can be considered a cold-tolerance strategy, while it is not the case under high-temperature stress since under high-temperature stress, flavonoid content in different plant organs may depict different patterns. ROSs’ scavenging properties of flavonoids are introduced as the functional role of flavonoids for both cold and heat stress, and membrane protection properties are proposed as a cold-stress alleviation strategy. Overall, the crosstalk of flavonoids with the various temperature stress response pathways is yet to be studied.

### 4.4. Atmospheric Pollutants

As biomarkers of air pollutants, the fluorescence emission of selected chloroplast metabolites, including flavonoids, carotenoids, lipofuscins, and pheophytins, revealed that nitric oxide (NO_2_) toxicity resulted in the modification of the fluorescence emission profile of carotenoids and flavonoids, suggesting a role for flavonoids in plants’ resistance against air pollutant stress [144]. HPLC analysis of the pollen grain of three ornamental plants grown under polluted areas contained mainly sulfur dioxide (SO_2_), NO_2_, carbon monoxide (CO), hydrocarbons (HC), and airborne particulate material (APM) revealed that the flavonoids content in ethanolic aquatic extracts of pollen grain of studied plants was increased. The increase in flavonoids led to reduced ROS accumulation in pollen grain and further resulted in improved pollen tube development and germination and eventually enhanced the plants’ fecundity [96]. Moreover, an antioxidative role is proposed for flavonoids in air pollutant stress scenarios. In this regard, it was noted that *Passiflora quadrangularis* L. plants grown in a hazy atmosphere synthesized more anthocyanin to cope with the oxidative stress caused by the hazy atmosphere [145]. In addition, an alternation in anthocyanin content has been noted in grape berry plants fumigated by SO_2_, which was claimed to be the result of preventing its degradation rather than de novo synthesis [146]. Application of H_2_S on *Brassica oleracea* L. resulted in an increase in anthocyanin content, which also accounts for the signaling role of H_2_S in antioxidative pathways [147]. Furthermore, treating *Vitis vinifera* cell suspension by the donor of H_2_S (sodium hydrosulfide) also resulted in increased flavonols and total phenolic, sinigrin, and anthocyanins [148].

Transcriptome and metabolome analysis of Malus crab apple indicated that a key (O_3_)-responsive transcription factor, McWRKY75, was positively correlated with a flavonoid-related structural gene. In addition, the exogenous application of methyl jasmonate decreased the negative impacts of O_3_ stress by enhancing the flavonoid metabolic pathway [149]. Studying *Medicago truncatula* response to O_3_ stress revealed that the potential for upregulation of flavonoid biosynthesis pathway and being benefited by flavonoids’ antioxidant properties account for the resilience of ozone-insensitive accession against O_3_ pollution [97]. Air pollutants intrude plant tissue through stomata and affect stomatal characteristics and apparatuses [150]. In cuticles and epicuticular waxes, flavonoids play the role of an antioxidant barrier to protect cellular components against air pollutants such as ozone (O_3_) and sulfur dioxide (SO_2_) [13].

Involvement in the signaling of stomatal movement and scavenging of ROS to block the transduction of signals that lead to stomatal malfunctioning is a well-defined role of flavonoids [112,115]. Given that, a possible role of flavonoids in ameliorating air pollutant stress can be their involvement in the signaling network of stomatal movement.

### 4.5. Light Stress

Light provides the fuel for photosynthesis, the process that a plant’s life entirely depends on. Light quality, intensity, and duration affect plant growth, morphology, resource acquisition, and adaption to the environmental condition [151,152,153,154]. Nevertheless, excess levels of light impose detrimental effects and cause light stress on plants. Flavonoids have been demonstrated to play a positive role in the amelioration of light stress effects on plants. However, some studies cast doubt on the positive role of flavonoids in stress response because, in some cases, flavonoids had a negligible role against light stress. Studies on different plant species showed that high light intensity increases flavonoid accumulation [155,156]. Further, the accumulation of flavonoids in epidermal cells, apical meristem, and pollens take part in filtering the extreme sunlight, thus reducing the likelihood of the collision of the harmful spectra on the vulnerable cellular organism causing oxidative stress [157]. Nevertheless, a contrasting report on the modification of flavonoid content under light stress is further elaborated by comprehensive metabolomics studies.

A study on *Ginkgo biloba* leaves exposed to UV-B radiation depicted a significant increase in the accumulation of flavonols in leaves under long-term UV-B exposure [158]. Similar work on white asparagus (*Asparagus officinalis* L.) showed that accumulation of a specific flavanol, quercetin-4′-O-monoglucoside, increased following exposure to UV-B stress [159]. Moreover, the effect of high light stress on the anthocyanin content of rose has been investigated and showed a high level of dependency on the light spectra in such a way that monochromatic red and blue light decreased while full-spectrum white light increased anthocyanin content. Interestingly, plants grown under white light depicted a better tolerance to high light stress [160]. Similarly, another study depicted that UV-B stress has a negligible effect on anthocyanin and flavonol index in cucumber plants grown under different light spectra [161]. Nevertheless, a more comprehensive metabolomics study showed that the ratio of four flavonoid compounds, kaempferol, quercetin, flavonol disaccharide I, and flavanol disaccharide II, varied after exposure to UV-B stress, and this modulation in flavonoids content was highly dependent on growing light spectra [162]. These reports suggest a spectral-dependent manner for the role of flavonoids in the regulation of light stress response.

### 4.6. Other Stresses

An *A. thaliana* ROS1-dependent flavonoid accumulation in response to herbicide stress has been revealed through the transcriptomic analysis of the imazethapyr-treated wild-type and ROS1 plants [163]. In an attempt to grow and develop multiple-herbicide resistance (MHR) in grass weeds, Schwarz et al. [164] examined the binding affinity of flavonoids to a phi class glutathione-S-transferase (AmGSTF1), which is a functional biomarker of MHR in black-grass (*Alopecurus myosuroides*). Using the ligand fishing experiment, they indicated that a variety of flavonoid structures are potent binders to AmGSTF1 [164].

It was indicated that stress caused by the flood could alter the accumulation pattern of flavonoids by influencing the expression of key enzymes involved in the flavonoid synthesis pathway and eventually resulting in an increase in the total flavonoid content of the *Chrysanthemum morifolium* [165]. The tolerance of *Pterocarya stenoptera*, a species widely distributed along rivers, to flooding stress was also attributed to increasing the synthesis of alpha-Linolenic acids and flavonoids in areal organs and activation of phytohormone biosynthesis and signaling pathways [166]. On the contrary, a study on soybean indicated that the genes related to the biosynthesis of phenylpropanoids, lignin, and flavonoids were downregulated under flooding stress and rendered plants’ roots more susceptible to pathogens [167]. These findings may propose the organ-specific response of flavonoid accumulation in plants under flooding stress.

## 5. Flavonoids-Mediated Abiotic Stress Signaling

**Figure 5:**
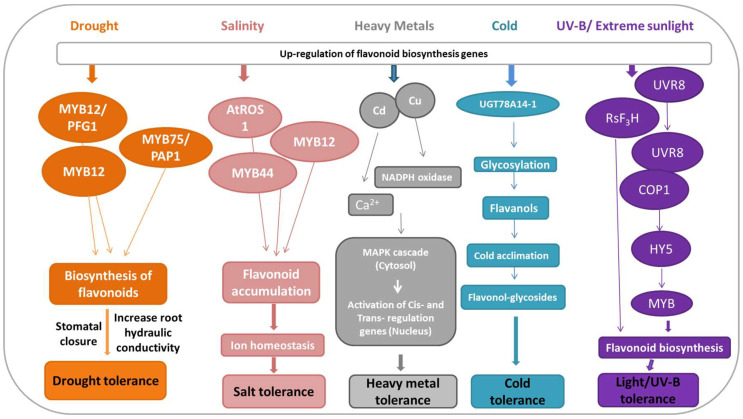
Actions of flavonoids under environmental stress conditions. Following abiotic stress, stress-specific transcription factors are activated and initiate the flavonoids biosynthesis pathway. Flavonoids improve plants’ resilience against abiotic stress by improving root hydraulic conductivity and stomatal movement under drought stress, improving ion homeostasis under salt stress, activation of cis- and trans-regulation genes in the nucleus under heavy metal stress, and increasing flavanol glycosides under cold stress.

Exposure of plants to external stresses initiates the increased regulation of flavonoid biosynthetic responsible genes, thus increasing the flavonoid content. In the desert plant *Reaumuria soongorica*, a rapid increase in RsF3H (flavanone 3-hydroxylase) gene expression and hindered lipid peroxidation triggered by antioxidant flavonoids has been evidenced as a protective strategy against UV-B and drought stress [168]. Tolerance to UV radiation occurs following flavonoids accumulation since they act as a sunscreen that filters the UV radiation, thereby hindering the generation of ROSs. The activation of UV-B photoreceptor activates the transcriptional factors (TFs), which further activate the transcription of flavonoid biosynthetic genes [13,169]. Similarly, UV-B stress in different species led to a modification in the transcription of flavonoid biosynthetic genes, which further enhanced the ratio of dihydroxy to monohydroxy B-ring-substituted flavonoid glycosides [170,171]. Luteolin and quercetin are glycosides actively involved in chelating iron (Fe) and copper (Cu) ions [56]. For instance, Berli et al. [172] observed that in grape leaves, UV-B radiation triggered an increase in quercetin derivates as an antioxidant for plant protection. It was demonstrated that when *A. thaliana* is exposed to drought stress, increased accumulation of flavonoids resulted in plant tolerance through the overexpression of MYB12/PFG1 (PRODUCTION OF FLAVONOL GLYCOSIDES1) or MYB75/PAP1 (PRODUCTION OF ANTHOCYANIN PIG-MENT1), MYB12 and PAP1, transparent testa4 (tt4) as a flavonoid-deficient mutant, and flavonoid-deficient MYB12 or PAP1 (obtained by crossing tt4 and the individual MYB overexpressor in *A. thaliana*) [2] Figure 5. In addition, direct estimation of the antioxidant activity revealed that enhanced accumulation of anthocyanin with effective in vitro antioxidant activity directly alleviates ROS in vivo [2]. In salt-stressed transgenic tobacco, overexpression of a repressor of silencing from Arabidopsis (AtROS1) occurred, which consists of genes encoding enzymes of flavonoid biosynthetic and antioxidant pathways, the influence of AtROS1 increasing the demethylation levels of these genes encoding CHS, CHI, F3-H, FLS, dihydroflavonol 4-reductase, and anthocyanidin synthase of the flavonoid biosynthetic pathway, and antioxidant enzymatic pathway that confirms the flavonoids mediated tolerance to salt stress [173]. Ismail et al. [174] reported that flavonoid (rutin) level increased by 25-fold in quinoa leaves under salt stress, which improved tissue tolerance and decreased the negative impact of high salinity on leaf photochemistry by elevating the availability of potassium (K^+^) and rate of (Na^+^) pumping. In addition, the negative correlation between rutin-stimulated modifications in K^+^ and H^+^ fluxes proposed that the accretion of rutin in the cytosol takes part in scavenging the hydroxyl radicals, thereby preventing K^+^ leakage through K^+^ efflux pathways [174]. These findings suggest the potential role of flavonoids in alleviating the negative impacts of abiotic stress.

## 6. Molecular and Genetic Approaches in Tailoring Flavonoids Biosynthesis and Regulation under Abiotic Stress

Several researchers adapted molecular techniques to examine the role of flavonoids in triggering the adaptive responses to abiotic stresses (Table 4). Calcium-dependent protein kinases actively participate in calcium signaling and stimulate the production of flavonoids to participate against the plethora of environmental stresses. In this regard, higher expression of GuCPKs genes in *Glycyrrhiza uralensis* under treatments of NaCl (30 mM) and CaCl_2_ (2.5 mM) has been reported. Induced expression of GuCPKs significantly improved the accumulation of flavonoid biosynthesis and glycyrrhizic acid under different salinity treatments [175]. Moreover, the study of Jan et al. [176] shows that transgenic plants with F3-H showed improved biosynthesis of quercetin and kaempferol in rice under salinity (150 mM) and heat stress (28–30 °C, light 16/8 h). They noted that heat and salinity stress increased oxidative damage, which was mitigated by the accumulation of flavonoid content. In addition, the overexpression of the AtMYB12 gene increased the accumulation of flavonoids by upregulating the genes involved in flavonoid biosynthesis in transgenic *Arabidopsis* under drought (25% PEG6000 for 2 weeks) and salinity (300 mM once every 2 days for 4 weeks) stresses [177]. Similarly, VvMyBF1 gene, cloned from grapevine, enhanced the accumulation of flavonoids in transgenic *Arabidopsis* for confronting drought (25% PEG6000 for 2 weeks) and salt stress (200 mM NaCl for 2 weeks) [178]. The transgenic plants showed higher activities of SOD, POD, pyrroline-5-carboxylate synthase, dihydroflavonol reductase, FLS, CHI, and PAL, as well as a significant reduction of MDA and H_2_O_2_ content. Overexpression of the GmMyB12 transcription factor increased the downstream flavonoids by improving the expression of flavonoid biosynthesis-related genes in *Arabidopsis* [179]. Its overexpression also increased the pyrroline-5-carboxylate synthase, SOD, and POD genes under salinity (200 mM NaCl, 2 weeks) and drought stress (25% PEG6000, 2 weeks). According to Wang et al. [180], a basic helix-loop-helix (bHLH) transcription factor gene antirrhinum (AmDEL) increased flavonoids accumulation under drought (25% PEG6000 for 2 weeks) and salinity (300 mM 2 days for 4 weeks) stresses via upregulating flavonoids biosynthesis genes in *Arabidopsis*. Moreover, the enzymatic analysis and Western blotting showed the higher activities of pyrrline-5-carboxylate synthase, dihydroflavonol reductase, CHI, and phenylalanine ammonia lyase (PAL) in transgenic plants as compared to wild plants against stressful conditions. Overexpression of SIbHLH22 in tomatoes showed small leaves, short height, and higher accumulation of flavonoids under drought (100 mM mannitol) and salt (200 mM NaCl) stresses [181]. Transgenic plants showed enhanced vigor by improving the ROS scavenging system. In another study, Jayaraman et al. [118] isolated gene encoding for chalcone isomerase 2 (OsCHI2) from drought-tolerant upland rice variety “Nagina22” and transduced it in drought-sensitive rice cv. Pusa Sugandh 2. by using inducible promotor AtRd29A. Stable chromosomal integration of transgenes showed abundant structural genes of flavonoid biosynthesis, which thereby resulted in higher production of flavonoids in mutant rice plants against abiotic stresses including heat (40 °C for 3 days), cold (2 °C; 16 h light/8 h dark for 12 days), salinity (150 mM NaCl for 7 days), and drought (withholding water 7 days at 9 to 10 leave stage) stresses. Their findings suggested that induction of OsCHI2 genes modulates flavonoid metabolism and enhanced abiotic stress tolerance, other than heat stress. In another case, the AeCHS gene isolated from *Abelmosschus esculentus* also increased flavonoid biosynthesis under treatments of osmotic (300 mM mannitol for a week) and salt (200 mM NaCl for a week) stresses in *Arabidopsis* plants [182]. Similarly, overexpression of the CHS gene in *Arabidopsis* improved high light stress by increasing the synthesis of anthocyanins that enhances the plant’s adaption to light when transferred from 100 µmol m^−2^ s^−1^ to 200 µmol m^−2^ s^−1^ [183].

Flavonol synthetase (FLS) is among the essential enzymes that participate in flavonoid biosynthesis. Wang et al. [184] overexpressed the *EkFLS* gene in *Arabidopsis*, isolated from *Euphorbia kansui* Liou under drought stress (20% PEG600) and salinity (200 mM NaCl) stresses. Their results revealed that *EkFLS* overexpression was strongly correlated with higher flavonoid biosynthesis and offer the theoretical basis for further improving the phytoextracts of medicinal plants and their resistance against multiple stresses simultaneously. Dong et al. [185] characterized *GSA1* (a quantitative trait locus regulating grain-size of rice) that encodes a UDP-glucosyltransferase and exhibits glucosyltransferase activity toward monolignols and flavonoids. They noted that *GSA1* redirects the metabolic flux from lignin synthesis toward flavonoid synthesis under abiotic stresses and accumulates more glycosides and flavonoids in rice for abiotic stress tolerance. Moreover, the *GAS1* overexpression resulted in larger grain size and played a key role in metabolic flux direction against multiple stresses, including salinity (150 mM for 7 days), drought (16% PEG8000 for 2 to 3 weeks), and heat (42 °C for dozens of h) stresses. Their findings suggested that *GSA1* catalyzes the glucosylation of flavonoids and monolignols to modulate the metabolic flux by altering the phenylpropanoid pathway and flavonoid glycoside profile in response to abiotic stress conditions. In another case, Li et al. [88] cloned the *Arabidopsis glycosyltransferase gene* (*UGT76E11*), and overexpressing plants showed substantially enhanced tolerance against H_2_O_2_ (0.4 mM), drought (200 mM mannitol), and salinity (100 mM NaCl for 10 days) stresses through producing higher glucosylate quercetin by modulating the flavonoid biosynthesis pathway as compared to wild plants. In another study, Li et al. [187] recognized two differentially expressed leucoanthocyanidin dioxygenase genes (*RtLDOX/RtLDOX2*) rapidly upregulated in *Reaumuria trigyna* under drought and salinity stress, consistent with stress-related cis-elements located in the promoter region. Transgenic *Arabidopsis* overexpressing *RtLDOX2* showed a higher accumulation of flavonols and anthocyanin, suggesting that this gene functions as a multifunctional dioxygenase in the flavonoid pathway and converts dihydrokaempferol to kaempferol. They noted that transgenic plants via agrobacterium-mediated transformation showed higher tolerance against drought (150 mM and 300 mM mannitol for 15 days), salinity (75 mM and 100 mM NaCl for 10 days), and ultraviolet-B (30 min per day for 7 days) stresses by modulating the flavonoid’s pathway and scavenging ROS.

## 7. Conclusions

Sessile plants develop various endogenous defense mechanisms to counter unfavorable conditions. Flavonoids are among the natural tools developed by plants to cope with abiotic stresses. This review encloses an overview of the functional roles of flavonoids in shaping a response to abiotic stress via the regulation of antioxidant systems, involvement in the signaling network, and modulation of physiological aspects of the plant. The biosynthesis of flavonoids and their accumulation in plants is triggered by abiotic stimuli and consequences in the modulation of stress response pathways. Flavonoids improve plants’ tolerance to abiotic stress at physiological and biochemical levels by the improvement of antioxidant capacity, regulation of cellular redox, activation of stress-responsive TFs, osmoregulation, and involvement in the stress response signaling network as a signaling molecule. We also discussed that flavonoids regulate stress response in different parts of plants, including, stomata, pollen grain, thylakoid membrane, cell membrane, and nucleus. This review provides the current status of flavonoids’ functional role in abiotic stress responses of plants and suggests flavonoids as a promising abiotic stress marker. Moreover, this review invites investigation of stress-specific flavonoids and the underlying exact mechanism of flavonoids’ involvement in stress responses, which can be a promising tool for crop breeding programs. Moreover, there are contrasting reports on the accumulation or reduction of flavonoid compounds in plants under light and temperature stresses. These remain to be delicately investigated using comprehensive methods such as metabolomics.

## Figures and Tables

**Table 4:** Molecular and genetic approaches in tailoring flavonoids biosynthesis and regulation under abiotic stress.

Genes, Transcript	Method	Plant Species	Stress	Results	Reference
*GuCPKs*	Induced expression of *GuCPKs* gene	*Glycyrrhiza uralensis*	NaCl (30 mM) and CaCl_2_ (2.5 mM)	Improved the accumulation of flavonoids biosynthesis and glycyrrhizic acid.	[175]
*flavanol 3-hydroxylase*.	Induced expression of *flavanol 3-hydroxylase* gene	Rice	Salinity (150 mM) and heat stress (28–30 °C, light 16/8 h)	Improved biosynthesis of quercetin and kaempferol. Increased oxidative damage, which was mitigated with the accumulation of flavonoids content.	[176]
*AtMYB12*	Overexpression of *AtMYB12*	*Arabidopsis*	Drought (25% PEG6000 for 2 weeks) and salinity stress (300 mM once every 2 days for 4 weeks)	Increased the flavonoids agglomeration by the upregulation of genes actively involved in flavonoid biosynthesis	[177]
*VvMyBF1*	*VvMyBF1* gene cloned from grapevine induced into Arabidopsis	*Arabidopsis*	Drought (25% PEG6000 for 2 weeks) and salt stress (200 mM NaCl for 2 weeks)	Increased the accumulation of flavonoids. Higher activities of SOD, POD, pyrroline-5-carboxylate synthase, dihydroflavonol reductase, FLS, CHI, and PAL, as well as a significant reduction of MDA and H_2_O_2_ content.	[178]
*GmMyB12*	Overexpression of *GmMyB12*	*Arabidopsis*	Salinity (200 mM NaCl, 2 weeks) and drought stress (25% PEG6000, 2 weeks)	Increased the downstream flavonoids by improving the expression of flavonoid biosynthesis-related genes. Increased the *pyrroline-5-carboxylate synthase*, *SOD*, *and POD.*	[179]
*Basic helix-loop-helix* (*bHLH*)	Transcription factor gene of (*bHLH*) *antirrhinum* (*AmDEL*) induced in Arabidopsis	Arabidopsis	Drought (25% PEG6000 for 2 weeks) and salinity stress (300 mM 2 days for 4 week)	Higher activities of pyrrline-5-carboxylate synthase, dihydroflavonol reductase, chalcone isomerase, and phenylalanine ammonia lyase (PAL) in transgenic plants. Upregulated flavonoids biosynthesis genes.	[180]
*SIbHLH22*	Overexpression of *SIbHLH22*	Tomato	Drought (100 mM mannitol) and slat stress (200 mM NaCl)	Transgenic plants showed enhanced vigor by improving ROS scavenging system. Showed small leaves, short height, and higher accumulation of flavonoids.	[181]
*Chalcone isomerase 2* (*OsCHI2*)	Induction of *OsCHI2*	Rice	Heat (40 °C for 3 days), cold stress (2 °C; 16 h light/8 h dark for 12 days), salinity stresses (150 mM NaCl for 7 days), and drought stress (withholding water 7 days at 9 to 10 leave stage).	Abundant structural genes of flavonoid biosynthesis and modulation of flavonoid metabolism.	[118]
*AeCHS* o in *Arabidopsis* plants	*AeCHS* gene isolated from *Abelmosschus esculentus* and induced in *Arabidopsis*.	*Arabidopsis*	Osmotic (300 mM mannitol for a week) and salt stress (200 mM NaCl for a week)	Increased flavonoid biosynthesis and abiotic stress tolerance.	[182]
*CHS* gene by	Overexpression of *CHS* gene in *Arabidopsis*	*Arabidopsis*	High light stress (200 µmol m^−2^ s^−1^)	Increased the synthesis of anthocyanins that enhance the adaptability of plants against light stress.	[183]
*EkFLS* gene	Overexpressed the *EkFLS* gene in *Arabidopsis*; isolated from *Euphorbia kansui* Liou	*Arabidopsis*	Drought stress (20% PEG600) and salinity stress (200 mM NaCl)	Increased flavonoids biosynthesis and gave a theoretical base for improving the phytoextracts of medicinal plants and their resistance against multiple stresses simultaneously.	[184]
*GSA1* gene	Overexpression of *GSA1* in rice	Rice	Salinity stress (150 mM for 7 days), drought stress (16% PEG8000 for 2 to 3 weeks), and heat stress (42 °C for dozens of hours)	Redirected the metabolic flux from lignin synthesis toward flavonoids synthesis. Accumulated more glycosides and flavonoids.	[185]
*glycosyltransferase gene* (*UGT76E11*)	Overexpression of *UGT76E1*	*Arabidopsis*	H_2_O_2_ (0.4 mM), drought (200 mM mannitol), and salinity (100 mM NaCl for 10 days) stress	Showed substantially enhanced tolerance through producing of higher glucosylate quercetin by modulating flavonoid biosynthesis pathway.	[88]
*RtLDOX/RtLDOX2*	Expressed leucoanthocyanidin dioxygenase genes (*RtLDOX/RtLDOX2*) of *Reaumuria trigyna* in *Arabidopsis*	*Arabidopsis*	Drought (150 mM and 300 mM mannitol for 15 days), salinity (75 mM and 100 mM NaCl for 10 days), and ultraviolet-B-stress (30 min per day for 7 days)	Overexpression of *RtLDOX2* showed a higher accumulation of flavonols and anthocyanin and converted dihydrokaempferol to kaempferol, scavenging ROS.	[186]
*UDP-sugar glycosyltransferase gene* (*CrUGT87A1*)	*CrUGT87A1* cloned form *Carex rigescens* in Arabidopsis	*Arabidopsis*	Salt stress (100 mM and 125 mM NaCl for 7 days)	Higher accumulation of antioxidants and flavonoids.	[61]
*R2R3-MYB* (*SbMYB2* and *SbMYB7*)	Overexpression of R*2R3-MYB* form *Scutellaria baicalensis* in tobacco	Tobacco	Salt stress (150 mM NaCl), drought (0.2 M mannitol), and ABA (100 µ M) for 3, 6, and 9 days, respectively	Higher fresh weight, lower flavonoid synthesis gene and antioxidants, and higher phenylpropanoid accumulation.	[187]
*PA1*-type *MYB* transcription factor (*MdMYBPA1*)	*MdbHLH33* directly binds to the cis element of the *MdMYBPA1* responsive to low temperature	Apple (*Malus x domestica*)	Low temperature (14 °C)	Responded to flavonoid biosynthesis by synthesizing anthocyanin from proanthocyanin.	[188]
*Ethylene insensitive 2* (*EIN2*)	Overexpression of *EIN2*	Rice	Cd stress (10 µM for 10 days)	Increased flavonoid and phenolics biosynthesis.	[189]
*Core apple autophagy-related gene* (*MdATG8i*)	Overexpression of *MdATG8i*	Apple	Drought (withholding water for 6 days)	Higher photosynthesis, amino acids, flavonoids, and antioxidant activities, lower ROS and oxidized and insoluble proteins, higher roots hydraulic conductivity, and improved water uptake.	[190]
*AvFLS*	*Apocynum venetum* gene overexpression in *AvFLS* induced in tobacco	Tobacco	Salinity stress (200 mM for 72 h)	Increased flavonoids synthesis, absorbed more K^+^, maintained Na^+^/K^+^ homeostasis, and increased K^+^/Na^+^ ratio.	[191]
*SbMYB8*	Overexpression of R*2R3-MYB* form *Scutellaria baicalensis* in tobacco	Tobacco	Salt stress (150 mM NaCl), drought (0.2 M mannitol), and ABA (100 µM) for 3, 6, and 9 days, respectively	Higher flavonoid biosynthesis and antioxidants, and improved tolerance against stress.	[192]

## Data Availability

Not applicable.
